# The Association of HER-2 Expression with Clinicopathological Characteristics and Clinical Outcomes in Patients with Localized Prostate Cancer After Radical Prostatectomy

**DOI:** 10.3390/diagnostics15212717

**Published:** 2025-10-27

**Authors:** Shuo Wang, Ruijian You, Xiao Yang, Peng Du, Yiqiang Liu, Yongpeng Ji, Qiang Zhao, Yudong Cao, Jinchao Ma, Yong Yang

**Affiliations:** 1 Key Laboratory of Carcinogenesis and Translational Research (Ministry of Education), Urological Department, Peking University Cancer Hospital & Institute, Beijing 100067, China; 2 Key Laboratory of Carcinogenesis and Translational Research (Ministry of Education), Department of Pathology, Peking University Cancer Hospital & Institute, Beijing 100067, China

**Keywords:** prostate cancer, biochemical recurrence, HER-2, radical prostatectomy

## Abstract

**Background/Objectives:** The purpose of this study was to investigate the association between HER-2 expression and clinicopathological characteristics, biochemical recurrence (BCR) rate, and BCR-free survival in localized prostate cancer (PCa) patients after radical prostatectomy (RP). **Methods:** Between January 2018 and December 2019, 44 patients with pathologically confirmed localized PCa who underwent RP were included in this study. According to the expressed level of HER-2 protein, patients were divided into four cohorts: cohort-1 (HER-2 0), cohort-2 (HER-2 1+ or 2+), cohort-3 (HER-2 0 or 1+), and cohort-4 (HER-2 2+); the clinicopathological and clinical outcomes were analyzed and compared between cohort-1 and cohort-2, and cohort-3 and cohort-4, respectively. Univariable and multivariable COX regression models and Kaplan–Meier curves were used to determine the association between HER-2 expression and clinicopathological outcomes, including Gleason score (GS), pathological T (pT) stage, positive surgical margins (PSM), and BCR-free survival, respectively. **Results:** The median follow-up time was 43 months (IQR 35–49). Among the 44 patients, 20 (45.5%) exhibited HER-2 immuno-reactivity, including 14 (31.8%) with HER-2 1+, 6 (13.64%) with HER-2 2+, and 0 (0%) with HER-2 3+ staining. The proportion of patients with PSM was significantly lower in the HER-2 0 group than in those with HER-2 1+ or 2+ (25.0% vs. 65.0%, *p* = 0.008). Multivariable logistics regression models revealed that HER-2 1+ or 2+ was an independent risk factor that was strongly associated with a higher proportion of PSM (OR, 2.69; 95% CI, 0.62–11.71, *p* = 0.042). A total of 18 (40.9%) patients experienced BCR after surgery, including 6 (25%) in cohort-1 and 12 (60.0%) in cohort-2 (*p* = 0.019), as well as 13 (34.2%) in cohort-3 and 5 (83.3%) in cohort-4 (*p* = 0.023). Kaplan–Meier analysis showed that patients in cohort-1 (HER-2 0) had significantly longer BCR-free survival than those in cohort-2 (HER-2 1+ or 2+) (*p* < 0.001), and those in cohort-3 had longer BCR-free survival than those in cohort-4 (*p* < 0.001). Furthermore, patients with PSM showed significantly shorter BCR-free survival compared to those with patients with negative surgical margins (NSM) (*p* = 0.005). Multivariable Cox regression analysis revealed that HER-2 1+, 2+ (HR, 17.00; 95% CI, 1.38–210.22, *p* < 0.001), HER-2 2+ (HR, 2.85; 95% CI, 1.23–3.25, *p* = 0.004), and PSM (HR, 6.12; 95% CI, 3.08–11.72, *p* = 0.007) were all significant independent predictors of BCR following surgery. **Conclusions:** HER-2 expression is a common phenomenon in PCa; nearly half of the proportion of localized PCa had HER-2 1+ or 2+, but the cases that expressed HER-2 3+ were rare. Cases with HER-2 1+ or 2+ were more likely to develop BCR compared with HER-2 0. The HER-2 1+ or 2+ expression was closely associated with a higher incidence of PSM and was an independent predictor of shorter BCR-free survival in patients with localized prostate cancer after radical prostatectomy.

## 1. Introduction

Prostate cancer (PCa) is one of the most common malignant tumors in the world. Many factors influence the clinicopathology characteristics and progression of PCa. In recent years, the pathways have been increasingly understood as a transmembrane tyrosine kinase receptor with high homology to the epidermal growth factor receptor. HER-2 protein overexpression is not common in normal tissue but has been found in a variety of epithelial malignancies, including breast, ovarian, bladder, gastric, and endometrial carcinoma [1]. Since Slamon et al. first reported the association of HER-2 overexpression and poor outcome of breast cancer, it has been considered to be a factor predicting the poor outcome of malignant tumors [2]. Most recently, several studies reported that HER-2 protein overexpression might be associated with the progression of PCa by activating the androgen receptor signal pathway. HER-2-dependent signaling may promote the development of PCa into castration-resistant prostate cancer (CRPC) [3]. And in CRPC, HER-2 is amplified or overexpressed in up to 60% of patients and is strongly correlated with the progression of the disease and shorter survival time [4]. Even so, most of the studies have focused on advanced PCa, and few studies have examined the role of HER-2 expression in localized PCa.

Given these considerations, we investigated whether expression of HER-2 was associated with a poor clinicopathological outcome, a high biochemical recurrence (BCR) rate, and short BCR-free survival in patients with localized PCa treated by radical prostatectomy (RP).

## 2. Materials and Methods

This is a retrospective study approved by the medical ethics review committee (protocol code 2019KT30).

### 2.1. Patients

In total, 44 localized PCa cases who underwent laparoscopic RP between January 2018 and December 2019 were retrospectively reviewed. None of these patients received neo-adjuvant therapy before RP and adjuvant therapy after RP until the detection of BCR. Preoperative clinical characteristics, including age, serum total PSA (tPSA) value, PSA free/total (f/t) value, total prostate volume (TPV), body mass index (BMI), and biopsy positive cores (BPC), were collected. Postoperative pathological and clinical outcomes, including Gleason score (GS), pathological T (pT) stage, positive surgical margins (PSM), and BCR, were also recorded. These variables were compared based on the level of HER-2 expressed. Data on risk factors that might relate to BCR, including HER-2, GS, PSM, and pT stage, were collected, and the associations of these factors with BCR and BCR-free survival time were subsequently analyzed.

### 2.2. Study Endpoints

The primary endpoint of this study was BCR-free survival after radical prostatectomy, defined as the time from surgery to the date of BCR or last follow-up. BCR was defined by at least two consecutive postoperative serum PSA levels ≥ 0.2 ng/mL, according to the American Urological Association guidelines.

The secondary endpoints included the following: (1) the BCR rate after RP, compared between different HER-2 expression groups; and (2) the association between HER-2 expression and adverse clinicopathological features, including PSM, pT stage, and GS.

### 2.3. Procedure

Patients suspected of having prostate cancer at our institute underwent ultrasound-guided 13-core transrectal prostatic biopsy. Serum tPSA value and PSA f/t values were collected just 1–2 days before surgery and at least 3 weeks after prostatic biopsy. Prior to surgery, an MRI and a PSMA PET-CT were performed to rule out metastasis to the bone, lymph nodes, or distant organ metastasis. Laparoscopic RP was performed at least 25 days after the initial biopsy. Extra-fascial radical prostatectomy was conducted through an extraperitoneal approach by skilled surgeons at our institute, following the established technique described by Walsh et al. [5]. All surgical specimens were assessed by a sophisticated pathologist at our institute; tissue samples were tested for HER-2 protein expression. HER-2 positivity was documented if the expression was observed on either the original diagnostic biopsy or the RP specimen. Postoperatively, serum tPSA and f/t values were measured every 1 to 3 months for follow-up purposes.

### 2.4. Variables

After radical prostatectomy, the prostate volume was estimated in cm^3^ using the modified ellipsoid formulation: Volume = 0.523 × length × width × height. Pathologic GS were recorded, and patients were staged according to the 2010 American Joint Committee on Cancer system (AJCC, pathologic stage T1–T4) [6]. Tumors were further classified into low-grade (GS ≤ 6), intermediate-grade (GS = 7), and high-grade (GS ≥ 8) groups following the grading component of the D’Amico risk classification [7]. Body mass index (BMI) was calculated as weight (kg)/height (m)^2^. BCR was defined by at least two consecutive serum tPSA levels ≥ 0.2 ng/mL according to the American Urological Association guidelines [8]. Data on time free from BCR was subsequently collected. Sections of the surgical specimen containing prostate cancer were selected for the determination of HER-2 expression. This was evaluated using the FDA-approved immunochemical kit HercepTest (DAKO, Carpinteria, CA, USA). The HER-2 immunostaining status was catalogized into 4 types: HER-2 0: membrane stain absent; HER-2 1+: greater than 10% of tumor cells present with barely perceptible staining; HER-2 2+: greater than 10% of tumor cells present with weak-to-moderate staining; and HER-2 3+: greater than 10% tumor cells present with strong staining [9]. Then, patients were divided into 4 sub-cohorts based on the expression status of HER-2. Cohort-1: HER-2 0; cohort-2: HER-2 1+, 2+, and 3+; cohort-3: HER-2 0, 1+; and cohort-4: HER-2 2+ and 3+. The clinicopathological factors were analyzed and compared between cohort-1 and cohort-2, and cohort-3 and cohort-4, respectively.

### 2.5. Statistical Analysis

Statistical analyses were performed using IBM SPSS Statistics for Windows, Version 20.0. Measurement data conforming to the normal distribution, analyzed by Shapiro–Wilk test, were presented as mean ± SD. An independent sample *t* test was used to evaluate the differences between continuous variables, while Chi-square tests were performed to examine categorical variables. Univariate and multivariate analysis binary logistic regression models were used to evaluate the association between HER-2 expression and adverse pathological events, which were all compared with the reference group (Ref). Kaplan-Meier analyses were generated to illustrate BCR-free survival according to the level of HER-2 expressed using the log-rank test, and the survival curves were described. Finally, a univariable and multivariable Cox regression model was used to identify the co-variables that influence BCR. All statistical tests were two-sided, and a *p*-value < 0.05 was considered statistically significant.

## 3. Result

### 3.1. Clinicopathologic Characteristics of the Entire Cohort

In total, 44 localized PCa patients were enrolled and analyzed in this study. Among them, 2 (4.5%) patients had GS ≤ 6, 22 (50%) patients had GS = 7, and 20 (45.5%) patients had GS ≥ 8. Pathologically, 26 (59.1%) patients were pT2, and 18 patients (40.9%) were pT3. PSM was observed in 19 patients (43.2%). The patients’ clinicopathologic demographics are summarized in Table 1.

### 3.2. Sub-Cohorts Divided by Levels of HER-2 Protein Expression

Tumors were classified based on HER-2 protein expression via immunohistochemistry. Of the 44 cases, 20 (45.5%) had HER-2 immuno-reactivity and 24 (54.5%) had non-HER-2 immuno-reactivity. Among the immuno-reactive cases, 14 (31.8%) showed HER-2 1+ staining, 6 (13.7%) showed HER-2 2+ staining, and 0 (0%) showed HER-2 3+ staining. All patients were subsequently divided into four sub-cohorts according to the level of HER-2 protein expression. A total of 24 cases (54.5%) with HER-2 0 and 20 cases (45.5%) with HER-2 1+ or 2+ were classified into cohort 1 and cohort 2, respectively. Additionally, 38 cases (86.4%) with HER-2 0 or 1+ and 6 cases (13.6%) with HER-2 2+ were classified into cohort 3 and cohort 4, respectively.

Initially, data were analyzed and compared between cohort-1 and cohort-2, and cohort-3 and cohort-4, respectively. The proportion of PSM was significantly lower in cohort-1 compared to cohort-2 (25.0% vs. 65.0%, *p* = 0.008)**.** However, the distribution of age, BMI, tPSA, f/t, TPV, BCP, GS, and pT stage did not show any significant differences. The TPV was significantly lower in cohort-3 compared with cohort-4 (32.72 ± 11.57 vs. 43.73 ± 8.49 mL, *p* = 0.004). No significant differences were observed for age, BMI, tPSA, f/t, BCP, GS, PSM, and pT stage in Table 1 and Table 2.

To assess the association between HER-2 1+, 2+, and PSM, we conducted both univariable and multivariable logistic regression analyses. The results indicated that HER-2 expression of 1+ or 2+ was an independent risk factor strongly associated with a higher proportion of PSM (OR, 2.69; 95% CI, 0.62–11.71, *p* = 0.042) after surgery, as shown in Table 3.

### 3.3. Factors Associated with BCR and BCR-Free Survival

**Figure 1:**
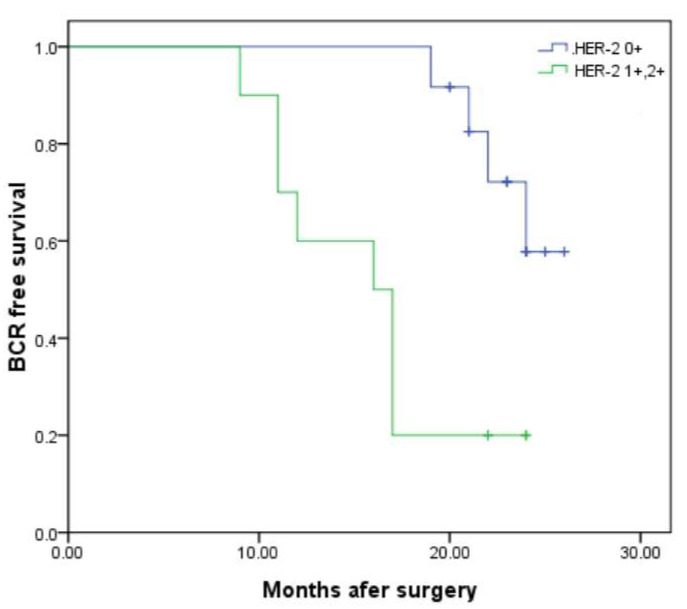
Kaplan–Meier curves for BCR-free survival according to expression level of HER-2. BCR-free survival of patients with HER-2 0 (cohort-1) was significantly longer than patients with HER-2 1+ or 2+ (cohort-2), 24.26 ± 0.513 vs. 15.8 ± 1.11 months; *p* = 0.000 by log-rank test.

**Figure 2:**
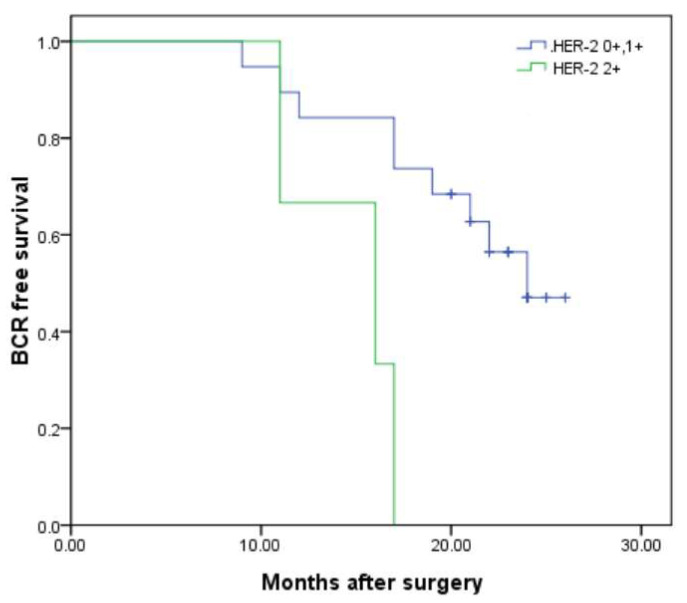
Kaplan–Meier curves for BCR-free survival according to HER-2 expression level. BCR-free survival of patients with HER-2 0 or 1+ (cohort-3) was significantly longer than patients with HER-2 2+ (cohort-4), 21.54 ± 0.92 vs. 14.67 ± 1.17 months; *p* = 0.000 by log-rank test.

The median follow-up time was 43 months (IQR 35.0–49.0). During this time, BCR was observed in 18 of 44 patients (40.9%). Factors associated with BCR, including HER-2 expression, GS, pT stage, and PSM, were collected and analyzed. The data revealed that 6 (25.0%) cases in cohort-1 and 12 (60.0%) in cohort-2 experienced BCR (*p* = 0.019). In cohort-3, 13 cases (34.2%) developed BCR, whereas in cohort-4, 5 (83.3%) cases developed BCR (*p* = 0.023). Furthermore, patients with PSM had a significantly higher rate of BCR than those with NSM (63.2% vs. 24.0%, *p* = 0.009). However, no significant differences were observed in the proportions of BCR between patients stratified by GS (*p* = 0.267) and pT stage (*p* = 0.15) (Table 4). Kaplan–Meier analysis demonstrated that patients with NSM had longer BCR-free survival compared to those with PSM (22.60 ± 1.25 vs. 14.622 ± 1.21 months, *p* = 0.005). Similarly, the BCR-free survival in cohort-1 was significantly longer in cohort-2 (24.26 ± 0.513 vs. 15.8 ± 1.11 months, *p* < 0.001) (Figure 1) and in cohort-3 than in cohort-4 (21.54 ± 0.92 vs. 14.67 ± 1.17 months, *p* < 0.001) (Figure 2). GS and pT stages were not associated with BCR-free survival. Univariable and multivariable Cox regression models revealed that HER-2 1+ or 2+ (HR, 17.00; 95% CI, 1.38–210.22, *p* < 0.001), HER-2 2+ (HR, 2.85; 95% CI, 1.23–3.25, *p* = 0.004), and PSM (HR, 6.12; 95% CI, 3.08–11.72, *p* = 0.007) were all identified as significant independent predictors of BCR. GS and pT stage were not independent prognostic factors of BCR (Table 5).

## 4. Discussion

This study was designed to evaluate the feasibility of molecular profiling in patients with localized PCa to determine the association between HER-2 protein expression and clinicopathological characteristics, as well as BCR following surgery. HER-2 oncoprotein is one of the four transmembrane receptors of the ErbB family. Upon ligand binding, it forms heterodimers with other ErbB family members to activate downstream signaling pathways [10]. HER-2 overexpression has been reported in various malignancies. For example, HER-2 overexpression is observed in approximately 30% of breast cancer cases. In patients with urothelial carcinoma of the bladder, HER-2 expression in circulating tumor cells was higher than in the corresponding primary tumor [11]. These findings suggested that HER-2-positive cells may have a greater capacity for vascular dissemination compared to their HER-2-negative counterparts.

A major clinical challenge in prostate cancer is its progression to an androgen-independent growth state following androgen ablation therapy. This transition is largely driven by the reactivation of the androgen receptor pathway, even in the absence of androgens [12]. However, the mechanism by which the pathway is activated in the absence of androgen is unknown. One important factor that may induce the development of androgen independence is the HER-2 oncoprotein. Preclinical studies suggest that HER-2 overexpression promotes AR transactivation and phosphorylation, potentially by enhancing the binding of AR to androgen-regulated gene promoters [3]. Furthermore, in vivo experiments have indicated that HER-2 is important for the survival of prostate cancer cells in an androgen-depleted environment [13]. Despite this mechanistic link, the reported prevalence of HER-2 overexpression in PCa varies across studies. For instance, one study reported that HER-2 overexpression in as high as 70% of localized PCa underwent radical prostatectomy [14]. In contrast, Koeppen et al. reported that only 5 of 61 PCa patients exhibited HER-2 overexpression classified as 2+, with none showing high-level (3+) expression [15]. Similarly, another study on hormone-refractory prostate carcinoma observed HER-2 overexpression in only 7% of patients [16]. Meanwhile, Yoshiaki et al. reported that 19.2% of the prostate cancer patients in M1b had HER-2 overexpression and had significantly poorer outcomes [17].

Our findings are consistent with the previous studies reported. In our study, 45.45% of cases had HER-2 expression, but only 13.64% of cases expressed HER-2 2+, and none expressed HER-2 3+. Patients with HER-2 1+ or 2+ expression had significantly higher rates of biochemical recurrence (BCR) and positive surgical margins (PSM), as well as shorter BCR-free survival, compared to those with HER-2 0. These findings indicate that even low-to-moderate HER-2 expression may have biological relevance in the early stages of PCa.

The observed association between HER-2 expression and a higher rate of PSM may be attributed to the biological behavior of HER-2-positive tumors. Activation of HER-2 triggers downstream MAPK and PI3K/AKT signaling pathways, promoting epithelial–mesenchymal transition (EMT), cell proliferation, and local invasiveness. Consequently, HER-2-positive tumors may more readily extend beyond the prostatic capsule or infiltrate periprostatic tissues, increasing the likelihood of positive margins following surgery. However, this association was not observed when comparing cohort-3 (HER-2 0/1+) and cohort-4 (HER-2 2+). This discrepancy may be explained by the limited sample size in the HER-2 2+ subgroup (*n* = 6), resulting in reduced statistical power. Additionally, both 1+ and 2+ represent low-to-moderate expression levels, and the biological difference between these categories may be insufficient to produce a measurable impact on surgical margin status in such a small cohort.

Several studies considered whether HER-2 overexpression may play an important role in the development of prostate cancer. One study obtained prostate cancer tissues from patients who were divided into three sub-cohorts: those treated with surgery alone (UNT), those treated with total androgen ablation therapy before surgery (TAA), and those treated with total androgen ablation therapy that failed and developed bone metastases (AI). The study found that HER-2 protein expression was significantly higher in TAA tumors than in UNT tumors. Additionally, the proportion of HER-2 positive tumors increased from UNT to TAA to AI. The authors concluded that HER-2 expression appears to increase with the progression to androgen independence [4]. Similarly, another study found that while the levels of HER-2 expression were infrequent in hormone-naïve tumors, they significantly increased in patients treated with androgen blockade [18]. HER-2 overexpression might be associated with the development of prostate cancer. In organ-confined prostate cancer, rare studies detected the relationship between HER-2 expression and clinicopathological characteristics. In 2008, one study investigated the expression of HER-2 and AR in organ-confined prostate cancer patients who underwent RP to determine whether alterations in these signal pathways contribute to the progression of the disease. The results showed that high expression of HER-2 and AR was both closely associated with a higher rate of PSA failure and a higher pathological stage [14]. Another study demonstrated that a higher level of HER-2 expression was associated with a higher prostate cancer stage and PSA progression in clinically localized PCa [19]. Our findings are partially consistent with the previous study. We also identified a significant association between HER-2 overexpression and poor outcomes; specifically, higher rates of BCR and PSM following RP. However, we did not observe a significant association with a higher GS and pT stage.

Given that HER-2 expression is a relatively common event in PCa patients and is implicated in the progression of the disease, targeting this pathway represents a promising strategy. Antibody–drug conjugates (ADCs) might be a potential medicine for treating PCa. ADCs are novel medicines consisting of a payload linked to specific antibodies that can recognize antigens expressed on cancer cells’ surfaces. Recently, ADC development in PCa has focused on targets like STEAP1, TROP2, PSMA, and CD46 [20,21]. The studies detecting the ADCs targeted on the HER-2 antigen are rare, with only a few cases being reported. Therefore, more research explorations are needed for detecting the applications of ADC targeting on HER-2 antigen.

This overlapping grouping in this study was an intentional exploratory analysis designed to explore different clinical cutoffs for HER-2 expression intensity, in order to maximize the pathological significance of HER-2 expression for prostate cancer prognosis. Cohort 1 (HER-2 0) vs. cohort 2 (HER-2 1+ or 2+): This comparison was designed to explore whether there was a prognostic difference at any level of HER-2 expression, i.e., whether it was positive (1+, 2+) versus negative (0). Our results confirmed that even the inclusion of low-level expression (1+) was sufficient to result in a higher risk of PSM and BCR in cohort 2. Cohort 3 (HER-2 0 or 1+) vs. cohort 4 (HER-2 2+): This comparison was designed to explore whether the highest level of expression (2+) was more clinically prognostic than low levels of expression (0 or 1+). In many cancer types, pathologists are concerned about whether high levels of expression (e.g., 2+ or 3+) represent more aggressive biological behavior. The simultaneous presentation of a comparison of these two different cutoffs will help provide a more comprehensive data foundation for future studies to determine the most predictive HER-2 expression cutoff. Therefore, the grouping of this study is exploratory, and due to the limited sample size, the results should be interpreted with caution.

Our study still has some limitations. First, it was a single-center, retrospective analysis with a relatively small sample size, which may limit the statistical power and generalizability of the findings. Second, HER-2 expression was evaluated by immunohistochemistry only, without confirmation by fluorescence in situ hybridization (FISH) or quantitative methods, which may introduce potential misclassification bias. Third, the absence of patients with HER-2 3+ expression precluded a full assessment of the prognostic value of high-level HER-2 positivity. Fourth, follow-up duration was moderate, and long-term oncologic outcomes, such as metastasis-free or overall survival, were not analyzed. Finally, external validation with larger, multicenter, prospective cohorts is warranted to confirm these preliminary observations.

## 5. Conclusions

The data indicated that nearly half of localized PCa exhibited HER-2 1+ or 2+, but the patients’ expressions of HER-2 3+ were rare. The HER-2 expression was significantly associated with a higher incidence of BCR and PSM and shorter BCR-free survival, but not associated with GS and pT stage. However, further well-designed and large-scale prospective studies are warranted to validate these findings.

## Figures and Tables

**Table 1:** Clinicopathological characteristics of the entire cohort and sub-cohorts divided by HER-2 expression.

	Entire Cohort	Cohort-1 (HER-2 0)	Cohort-2 (HER-2 1+, 2+)	*p* Value
Number	44	24	20	
Age (years)	67.95 ± 6.89	68.33 ± 7.70	67.5 ± 5.93	0.694
BMI (kg/m^2^)	25.60 ± 2.13	25.51 ± 2.25	25.70 ± 2.02	0.771
tPSA (ng/mL)	29.49 ± 40.59	36.66 ± 51.68	20.89 ± 18.80	0.203
f/t	0.129 ± 0.01	0.119 ± 0.047	0.148 ± 0.046	0.177
TPV (mL)	34.77 ± 12.28	32.82 ± 11.09	37.11 ± 13.48	0.254
BCP (%)	44.65 ± 22.48	49.03 ± 17.91	39.01 ± 26.93	0.216
GS (*n*, %)				0.40
≤6	2 (4.5)	2 (8.3)	0 (0)	
7	22 (50)	12 (50)	10 (50)	
≥8	20 (45.45)	10 (41.7)	10 (50)	
pT stage (*n*, %)				0.911
pT2	26 (59.09)	14 (58.3)	12 (60)	
pT3	18 (40.91)	10 (41.7)	8 (40)	
PSM (*n*, %)	19 (43.18)	6 (25)	13 (65)	0.008

BMI: body mass index; tPSA: total prostate cancer-specific antigen; TPV: total prostate volume; BCP: biopsy core positive; GS: Gleason score; pT: pathological stage; PSM: positive surgical margins; BCR: biochemical recurrence.

**Table 2:** Clinicopathological characteristics of patients divided by HER-2 expression.

	Cohort-3	Cohort-4	*p* Value
	(HER-2 0, 1+)	(HER-2 2+)	
Number	38	6	
Age (years)	67.79 ± 7.14	69.00 ± 5.44	0.694
BMI (kg/m^2^)	25.53 ± 2.12	26.04 ± 2.34	0.588
tPSA (ng/mL)	31.25 ± 43.36	12.46 ± 0.50	0.299
f/t	0.129 ± 0.048	0.113 ± 0.069	0.521
TPV (mL)	32.72 ± 11.57	43.73 ± 8.49	0.004
BCP (%)	49.84 ± 18.86	29.37 ± 32.58	0.043
GS (*n*, %)			0.499
≤6	2 (5.3)	0 (0)	
7	20 (52.6)	2 (33.3)	
≥8	16 (42.1)	4 (66.7)	
pT stage (*n*, %)			1
pT2	22 (57.9)	4 (66.7)	
pT3	16 (42.1)	2 (33.3)	
PSM (*n*, %)	15 (39.47)	4 (66.7)	0.211

BMI: body mass index; tPSA: total prostate cancer-specific antigen; TPV: total prostate volume; BCP: biopsy core positive; GS: Gleason score; pT: pathological stage; PSM: positive surgical margins; BCR: biochemical recurrence.

**Table 3:** Univariable and multivariable analysis of the impact of HER-2 expression on status of surgical margins.

	Univariable Analysis			Multivariable Analysis		
	PSM vs. NSM			PSM vs. NSM		
	HR	95% CI	*p* Value	HR	95% CI	*p* Value
HER-2 0	Ref	Ref		Ref	Ref	
HER-2 1+, 2+	2.333	0.897–6.072	0.046	2.691	0.619–11.71	0.042
HER-2 0, 1+	Ref	Ref		Ref	Ref	
HER-2 2+	1.25	0.625–11.233	0.082	2.667	0.298–23.858	0.380

Ref: reference; PSM: positive surgical margins; NSM: negative surgical margins.

**Table 4:** Clinicopathological characteristics of the patients divided by status of BCR.

	No BCR 26	BCR 18	*p* Value
HER-2 0 (*n*, %)	18 (75)	6 (25)	0.019
HER-2 1+, 2+	8 (40)	12 (60)	
HER-2 0, 1+ (*n*, %)	25 (65.79)	13 (34.21)	0.023
HER-2 2+	1 (16.67)	5 (83.33)	
GS (*n*, %)			0.151
≤6	2 (10)	0 (0)	
7	15 (50)	7 (50)	
≥8	9 (40)	11 (50)	
pT stage (*n*, %)			0.100
pT2	18 (69.23)	8 (30.77)	
pT3	8 (44.44)	10 (55.56)	
PSM (*n*, %)	7 (36.84)	12 (63.16)	0.009
NSM (*n*, %)	19 (76)	6 (24)	

PSM: positive surgical margins; NSM: negative surgical margins; GS: Gleason score; pT stage: pathological T stage.

**Table 5:** Univariate and multivariable Cox regression analysis for BCR-free survival based on two distinct HER-2 expression definitions.

	Univariable Analysis			Multivariable Analysis		
	HR	95% CI	*p* Value	HR	95% CI	*p* Value
HER-2 0	Ref	Ref		Ref	Ref	
HER-2 1+, 2+	4	1.337–11.965	0.005	17.002	1.378–210.216	<0.001
HER-2 0, 1+	Ref	Ref		Ref	Ref	
HER-2 2+	1.615	0.825–2.424	0.015	2.849	1.234–3.246	0.004
GS						
≤6	Ref	Ref		Ref	Ref	
7	1.5	0.613–3.670	0.374	8.745	0.535–2.822	0.151
≥8	1.25	0.493–3.167	0.638	5.441	0.789–2.632	0.452
Surgical margin						
NSM	Ref	Ref		Ref	Ref	
PSM	6.5	3.377–12.51	0.006	6.118	3.083–11.72	0.007
pT stage						
T2	Ref	Ref		Ref	Ref	
T3	1.167	0.540–2.522	0.334	17.022	0.678–2.216	0.327

Table 5 presents results from two separate Cox regression models. Model 1 includes HER-2 expression defined as 0 vs. 1+ or 2+. Model 2 includes HER-2 expression defined as 0 or 1+ vs. 2+. Both models are presented for exploratory purposes to investigate the prognostic value of different HER-2 expression thresholds. GS: Gleason score; PSM: positive surgical margins; NSM: negative surgical margins; pT stage: pathological T stage; Ref: reference.

## Data Availability

The data presented in this study are available on request from the corresponding author due to the data involve institutional confidential information regulated by the national health administration (e.g., internal hospital management data, regional public health statistics), which are not permitted to be publicly shared per official policies.
